# Effects of Different Crystalloid Fluids on Renal Tissue in an Experimental Model of Hemorrhagic Shock

**DOI:** 10.4274/TJAR.2023.231262

**Published:** 2023-10-24

**Authors:** Kemal Tolga Saraçoğlu, Ayten Saraçoğlu, Mehmet Yıldırım, Cumaali Demirtaş, Metehan Akça, Ferda Serdoğan, İlyas Samet Ergün, Şermin Tetik, Sadrettin Pençe

**Affiliations:** 1 Department of Anaesthesiology, ICU & Perioperative Medicine, Hazm Mebaireek General Hospital HMC, Doha, Qatar; 2 Qatar University College of Medicine, Doha, Qatar; 3 Department of Anaesthesiology, ICU & Perioperative Medicine, Aisha Bint Hamad Al Attiyah Hospital HMC, Doha, Qatar; 4 Department of Physiology, Hamidiye Faculty of Medicine, University of Health Sciences Turkey, İstanbul, Turkey; 5 Department of Physiology, Faculty of Medicine Tokat Gaziosmanpaşa University, Tokat, Turkey; 6 Clinic of Anaesthesiology and Intensive Care, Turhal State Hospital, Tokat, Turkey; 7 Department of Medical Services and Techniques, Bezmialem Medical School, İstanbul, Turkey; 8 European University of Lefke, Faculty of Pharmacy Lefke, Northern Cyprus TR-10 Mersin, Turkey; 9 Department of Physilogy, İstanbul Medeniyet University, İstanbul, Turkey

**Keywords:** Annexin A5, hemorrhagic shock, normal saline, Ringer’s lactate, ROTEM

## Abstract

**Objective:**

The type of fluid that should be used in uncontrollable hemorrhages remains an area of research. This study was designed to compare the effects of resuscitation with Ringer’s lactate (RL) solution versus a normal saline (NS) solution on hemodynamics, renal tissue histopathology, coagulation, and apoptosis in a rat model of hemorrhagic shock.

**Methods:**

The study employed groups designated as the control, hemorrhage, NS, and RL groups. Heart rate, mean arterial pressure, and respiratory rate were monitored. Annexin A5 values were assayed, rotational thromboelastometry analysis was performed, and excised kidney tissue samples were histopathologically analyzed.

**Results:**

Blood pressure levels were found to be significantly higher in the control group than those measured in the other groups. While the clotting time (CT) and clot formation time (CFT) in the hemorrhage group were significantly longer than those in the control and RL groups, the CT and CFT measured in the control group were significantly shorter compared to the RL group. The mean Annexin A5 level was in the hemorrhage group, which was significantly higher compared to the other groups. In the renal histopathological evaluation, the scores of proximal tubular injury, distal renal tubular injury, and interstitial renal tubular injury were found to be significantly lower in the control group compared to the other groups.

**Conclusion:**

This study demonstrated that NS or RL can be used safely to improve the hemodynamic symptoms resulting from hemorrhagic shock as a means to reduce apoptosis, and to decrease findings in favor of coagulopathy in bedside coagulation tests during the early stages of hemorrhagic shock until the time of starting a blood transfusion.

**Main Points**

• Significant hypotension and tachycardia occur following hemorrhagic shock and clot formation is delayed as trauma-induced coagulopathy intensifies and hypotension deepens.

• Traumatic hemorrhage causes microcirculatory alterations resulting in histopathological injury and apoptosis in renal tissue.

• Normal saline and Ringer’s lactate can be safely used in the early phase of traumatic hemorrhagic shock during the time till blood is available to start transfusion.

## Introduction

The most common cause of hypovolemic shock includes traumatic hemorrhagic shock triggered by soft tissue damage, as well as an immune response in addition to acute hemorrhage.^1^ Trauma, which is the leading cause of preventable deaths, still results in high mortality rates due to life-threatening and uncontrollable hemorrhaging.^2^ The aim of damage control resuscitation, an aspect of damage control surgery introduced to prevent such deaths, is to maximize tissue oxygenation by achieving the targets of hemostasis, homeostasis, and hemodynamics, and to control bleeding.^3^ According to a recently published systematic review, no comparative study has to date investigated the effects of these fluid types on microcirculation.^4^

This study was designed to compare the effects of resuscitation using a Ringer’s lactate (RL) solution versus normal saline (NS) on hemodynamics, renal tissue histopathology, coagulation, and apoptosis in a rat model of hemorrhagic shock.

## Methods

This experimental trial was approved by the Animal Experimentation Ethics Committee at University of Health Sciences Turkey (protocol number: 46418926-605.02; date: 27.06.2019; chairperson: Prof. Dr. Sadrettin Pençe). Twenty 6-month-old Wistar rats were used in this trial. No preliminary study was performed leading to the death of animals. Rats were randomized into 4 groups and each group included 5 experimental animals.

### Allocation to Groups

Group C (control group): The group to which hemorrhage was not applied (n = 5).

Group H (hemorrhage group): The group to which hemorrhage was applied but in which rats did not receive any therapy (n = 5).

Group NS (normal saline group): The group to which hemorrhage was applied and in which rats were given intravenous (IV) NS (n = 5).

Group RL (Ringer’s lactate group): The group to which hemorrhage was applied and in which rats were given an infusion of RL (n = 5).

Following a 1-hour period, blood samples were collected to observe the effects of hemorrhagic shock at the tissue level. Then, crystalloid fluid was infused into the rats.

### Selection and Preparation of Animals

Male Sprague-Dawley rats weighing 214-268 g were used after 5-7 days of acclimatization. Rats were maintained in separate cages for 12 h during the day and 12 h at night. The ambient temperature was controlled.

### Anaesthesia Protocol

To avoid expected or unexpected side effects related to anaesthesia, the determined anaesthesia method was carefully applied to the animals by an experienced anaesthesiologist. The depth of anaesthesia was controlled by the pinch reflex. To correct the hypothermic state of the animals under anaesthesia, a homoeothermic blanket maintaining a constant body temperature at 37 °C was used. Rats received an intraperitoneal injection of ketamine hydrochloride at a dose of 90 mg kg^-1^ and xylazine hydrochloride at a dose of 10 mg kg^-1^ for anaesthesia.

### Monitoring

Hemoglobin oxygen saturation, respiratory rate per minute, heart rate, and core temperature were measured noninvasively and recorded. Blood pressure, respiratory rate, and heart rate were monitored using an electrophysiological data acquisition system (PowerLab 16/35, AD Instruments, Castle Hill, Australia). Blood pressure was measured at the carotid artery with a 24-catheter employing a reusable BP transducer (MLT0380/D, AD Instruments, Castle Hill, Australia). The carotid artery was also used to collect blood samples and to generate hemorrhage. The tail vein of rats was cannulated for IV fluid infusion. Respiratory rate and heart rate were measured non-invasively and recorded. The respiration rate was measured using PowerLab-connected pulse transducers (TN1012/ST, AD Instruments, Castle Hill, Australia). Heart rate was measured using PowerLab-connected biological amplifiers (Bio Amp FE231, AD Instruments, Castle Hill, Australia).

### Protocol for Hemorrhagic Shock

The hemorrhagic shock protocol^5^ was initiated 60 min after all rats had been anaesthetized. The target blood volume loss was determined as 40%. Hemorrhage was performed in three stages. In the first stage, a 25% loss was achieved and the hemorrhage rate was 0.5 mL min^-1^. In the second stage, 10% blood loss was achieved at a rate of 0.2 mL min^-1^. In the third and final stage, 5% blood loss was achieved at a rate of 0.1 mL min^-1^. Hemorrhage was achieved in rats using a double-lumen femoral vein catheter. While blood was drawn from one lumen with a syringe, a sterile sodium citrate solution was administered through the other lumen. The procedure was interrupted in a case when the mean arterial pressure fell below 40 mmHg. Later, hemorrhage was continued when a pressure of 45 mmHg was maintained for more than 15 s. The hemorrhagic shock protocol was applied for a total period of 60 min, then IV crystalloid infusions were started in the study groups.

Rotational thromboelastometry (ROTEM), Annexin A5, and lactate levels were recorded at T0 and T1 as follows:

**T0:** The time when the blood sample was taken before the initiation of hemorrhage (baseline measurement).

**T1:** The time when the blood sample was taken after resuscitation.

At the end of the procedures, the vascular catheters were removed, the incisions were closed and the rats were sacrificed. Finally, tissue samples were collected and examined.

### Resuscitation Fluids

Animals were randomized to receive RL or NS three times the volume of blood lost. Resuscitation fluids were heated to 37 °C and infused within 30 min. Plasma obtained from blood samples and collected in citrated tubes was stored at -80 °C and used for immunoassay methods. Samples collected for blood gas analysis were kept in a heparinized tube until analysis.

### Rotational Thromboelastometry

Ninety cartridges were used for the ROTEM^6,7^ procedure. Of the parameters evaluated for ROTEM, clot formation time (CFT) represents the time (min) from a clot amplitude of 2 mm until a clot amplitude of 20 mm is achieved. Maximum clot firmness (MCF) represents the point where the clot reaches its highest strength and firmness. A10 represents the clot amplitude at 10 min of clotting.

### Renal Histopathological Evaluation

Kidney tissue samples were collected and fixed with a 10% neutral buffered formalin solution and embedded in paraffin. Serial sections of 4-5 µm thick paraffin blocks were obtained with Thermo microtome, and the sections were hematoxylin and eosin-stained. The sections were photographed using a Leica DM2000 light microscope and with the camera of the iPhone X mobile phone. Sections were evaluated for proximal and distal tubular injury, glomerular injury, and interstitial and vascular injury.

### Scoring Used for Assessment of Kidney Injury

### Monitoring Endothelial Injury, Apoptosis, and Coagulation Using the Enzyme-Linked Immunosorbent Assay Method

Annexin 5 measurements were performed using enzyme-linked immunosorbent assay (ELISA) kits. To obtain serum, blood samples (5 mL) were collected into a tube containing vacutainer serum separator gel, and the tubes were kept at room temperature and centrifuged at 100x g for 10 min within the first hour of blood collection. After separation, the serums were frozen at -20 °C, and Annexin A5 measurements were performed using ELISA kits.

### Statistical Analysis

The SPSS Statistics 22.0 software was used for the analyses. We employed the descriptive statistics of mean, standard deviation, median, minimum, maximum, frequency, and ratio. The Kolmogorov-Smirnov test was used to measure the distribution of the variables. Quantitative independent data were analyzed using the Mann-Whitney U test. Dependent quantitative data was analyzed using the Wilcoxon test. For the analysis of repeated parameters, repeated measure analysis of variance (ANOVA) was used. Quantitative independent data were analyzed using a chi-square test, but if the conditions for this test were not met, we used the Fisher test. A one-way ANOVA test and the post-hoc Tukey test were used for normally distributed numerical data.

## Results

### Vital Parameters

The “respiratory rate at min 60” was statistically significantly lower in the control group than in the NS group (*P*=0.049, Table 1). The respiratory rates of rats during hemorrhage were similar between the groups (Figure 1).

The “heart rate measured at min 60 after hemorrhage” was found to be statistically significantly higher in the control group compared to the hemorrhage and NS groups (*P*=0.014). The heart rates measured at the “0 and 30 min of crystalloid infusion” were statistically significantly higher in the control group than in group H (*P* < 0.05, Table 2).

“Blood pressure levels measured at the “10 and 15 min of hemorrhage” and at “0 min of infusion” were found to be statistically significantly higher in the control group than those measured in the other groups (*P*=0.001, Table 3). The blood pressure values measured at “30 and 60 min of the crystalloid infusion” in the hemorrhage group were found to be statistically significantly lower than the measurements in the C, NS, and RL groups (*P*=0.001). The blood pressure values measured at “10 min of the crystalloid infusion” in the control group were found to be statistically significantly higher compared to the same values in the H and RL groups, while it was statistically significantly higher in the NS compared to the H group (*P* < 0.05). The blood pressure value measured at “15 min of infusion” was statistically significantly lower in the hemorrhage group than the same value recorded in the C, NS, and RL groups, whereas it was found to be statistically significantly lower in RL compared to the C group (*P* < 0.05, Table 3).

### ROTEM Analysis

The CT and CFT values in group H were statistically significantly higher compared to the other groups (*P*=0.001). A10 measurement in group H was statistically significantly lower compared to the other groups (*P*=0.003). The MCF value measured in group H was found to be statistically significantly lower than those in the NS and RL groups (*P*=0.001, Table 4).

While CT and CFT in group H were statistically significantly longer than those in groups C and RL, the CT and CFT measured in group C were statistically significantly shorter compared to the RL group (*P*=0.001). A10 measurement in group H was statistically significantly lower compared to group C (*P*=0.015). Group C had a statistically significantly higher MCF value compared to those measured in the H and RL groups (*P*=0.001, Table 5). A10 and MCF measurements in the hemorrhage group were observed to be statistically significantly lower compared to the RL group (*P* < 0.05).

A10 and MCF measurements for the hemorrhage group in FIBTEM were observed to be statistically significantly lower compared to the RL group (*P* < 0.05, Table 4).

The CT measured in group H was found to be statistically significantly higher than the values measured in the K and RL groups (*P*=0.001, Table 4).

### ELISA Analysis

The mean Annexin A5 level was 16.36±4.47 ng mL^-1^ in the hemorrhage group, which was statistically significantly higher compared to the other groups (*P*=0.002).

### Histopathological Evaluation

In the renal histopathological evaluation, the groups were compared in terms of proximal, distal, and interstitial tubular injury scores. These scores were found to be significantly lower in the control group compared to those in the other groups (*P *< 0.05, Table 5). There was no significant difference between the NS and RL groups regarding proximal, distal, and interstitial renal tubular injuries (*P *> 0.05). No significant difference was observed when the proximal renal tubular injury scores of the hemorrhage group were compared to those of the NS and RL groups (*P*=0.549 and *P*=0.062, respectively). We did not observe any significant difference when the proximal renal tubular injury scores of the hemorrhage group were compared to those of the NS and RL groups (*P*=0.0766 and *P*=0.172, respectively). The interstitial renal tubular injury scores of the hemorrhage group were compared to those of the NS and RL groups, and no significant difference was detected (*P*=0.208 and *P*=0.135, respectively).

### Correlation Analysis

A positive correlation was found between the value obtained from Annexin A5 measurement and the measurements of CT Extem, CFT Extem, CT Intem, CFT Intem, CT Heptem, and that of proximal tubular injury (*P *< 0.05). Furthermore, a negative correlation was observed between A10 Extem, MCF Extem, A10 Intem, MCF Intem, A10 Fibtem, MCF Fibtem, and the blood pressure levels measured at min 0, 10, 15, 30, and 60 of crystalloid infusion (*P *< 0.05).

## Discussion

This experimental study revealed that significant hypotension and tachycardia occurred in rats following traumatic hemorrhagic shock induced by a constant volume-controlled bleeding model; additionally, as trauma-induced coagulopathy intensified and hypotension deepened, the initiation of CF was delayed. It was also observed that traumatic hemorrhage caused microcirculatory alterations, resulting in histopathological injury and apoptosis in the renal tissue. This study, in which NS and RL were compared, revealed that both crystalloids had corrective effects on the signs of shock, the development of apoptosis, CT, and the firmness, amplitude, and strength of the clot.

The selection of IV resuscitation fluid in hemorrhagic shock has been a topic of debate for more than a century. Normal saline and RL are administered as equivalent resuscitation fluids in many trauma centers. In our study, tachycardia secondary to hemorrhage was reduced more effectively in the NS compared to the RL group. There was a significant decrease in heart rate at the first hour of hemorrhage, and NS increased the blood pressure significantly at min 10 of the infusion compared to the hemorrhage group. It was also observed that an IV infusion of NS achieved a greater increase in mean arterial pressure compared to RL. Although the difference was not statistically significant, we believe that this to have been due to the small sample size and that increasing the number of samples would render the result statistically significant. We believe that this positive effect of NS in trauma patients may be due to the vasodilator effects resulting from the drop NS achieved in peripheral resistance on arterial blood pressure. Similarly, in a study comparing NS and RL on 20 swine that were given anesthesia, NS provided an increase in blood pressure.^8^ Moreover, better oxygen delivery has been also reported in the NS groups. A study previously conducted reported that swine that were given NS developed hyperchloremic metabolic acidosis, leading to coagulation disorders.^9^ However, in the same study, the volume of crystalloid infusion was not kept constant, and the volume of infused NS reached twice that of RL. However, another study reported that although NS caused the development of hyperchloremic metabolic acidosis, this condition did not lead to coagulation disorder according to ROTEM analyses.^10^ The main triggering factors in trauma-induced coagulopathy are coagulation pathway disorders, infection, and global disturbance of hemostasis caused by cellular dysfunction.^11^ When this happens, excessive fluid resuscitation leads to dilutional coagulopathy, increasing hemorrhage. In our study, the coagulation profile was monitored with ROTEM, which enabled the accuracy of the data to be objectively proven. The values of CT, CFT, and A provided information about clotting time and the firmness and amplitude of the clot. These values were observed to be impaired in the hemorrhage group. However, there was an improvement in the coagulation parameters in the NS and RL groups compared to the hemorrhage group, and even values close to those of the controls were also obtained.

The MCF measurement values provide information on the firmness of the clot. The MCF and A10 values in this study were found to be significantly lower in the hemorrhage group, whereas these indicated an increase in both the RL and NS groups. Replacing the IV volume at the appropriate time can likely prevent the formation of coagulopathy by preventing damage to the endothelial glycocalyx and impaired perfusion. As such, it becomes possible to prevent dilutional coagulopathy by keeping the amount of fluid infused limited. One of the important characteristics of our study is that both NS and RL were administered in equal amounts and in a manner that enabled exactly meeting the amount of blood loss when needed. Thus, we were able to make an effective comparison between NS and RL. The data of our study showed that neither crystalloid caused deterioration in coagulation, provided that they were not administered in excessive amounts or in an uncontrolled manner. We believe this data to be a significant contribution to the management of trauma patients in the pre-hospital setting.

Comparing the histopathological evaluation, our study shows that the damage following hemorrhage caused injury to the proximal and distal tubules and the interstitium. It was observed that damage at the microcirculatory level could not be corrected in rats infused with a crystalloid. The NS and RL groups did not indicate any advantage over one another in this regard.

The induction of apoptosis is an area of concern in RL administration.^12^ A study by Rhee et al.^13^ showed that RL infusion following hemorrhagic shock caused increased apoptosis in the intestinal mucosa, smooth muscles, as well as the cells in the liver and lungs. However, no significant apoptosis was observed in fluid resuscitation procedures performed using sham, plasma, fresh blood, and hypertonic saline. The infusion of RL led to increased adhesion molecule expression, causing amplified neutrophil activation and the release of reactive oxygen species. It has been suggested that this is how apoptosis progresses. It was reported in another study analyzing a hemorrhagic shock model in 49 Yorkshire swine that RL infusion caused hepatic and pulmonary apoptosis.^14^ However, there is very limited data in the literature regarding apoptosis in renal tissue. In our study, the mean Annexin A5 level was 16.36±4.47 ng mL^-1^ in the hemorrhage group, which was found to be statistically significantly higher compared to the other groups. This result indicates the presence of severe apoptosis during hemorrhage. Nevertheless, apoptosis significantly decreased in the groups that were administered RL and NS and even regressed to values close to those of the control group. On the other hand, early crystalloid infusion performed using RL or NS cannot prevent microcirculatory disorder. However, crystalloid infusion with RL or NS is promising in that it was shown to have an inhibitory effect on apoptosis. It has been demonstrated that coagulopathy and delayed CF due to traumatic hemorrhagic shock accelerate apoptosis. According to the data obtained in our study, as the coagulopathy intensified, hypotension deepened and an inversely proportional result was obtained with the onset of CF.

An RL solution is considered a balanced crystalloid mixture; conversely, NS solutions have different electrolyte contents and are associated with a risk of hyperchloremic metabolic acidosis when administered in high volumes. As a result, there is a risk of developing acute kidney injury.^15^ Accordingly, it is believed that the clinical results of IV infusions may differ. However, there are not enough comparative studies to prove this assumption. In this context, our research is among the studies that have aimed to clarify this controversial issue, as it indicated that both solutions could be safely used in the emergency department. Similarly, a study compared RL and NS in non-critically ill adults treated with IV fluids in the emergency department.^16^ The researchers administered an average of 1,079 mL of crystalloid fluid to 13,347 patients and found no difference in terms of length of hospital stay, which is closely related to patient prognosis. Another study compared IV NS and RL infusions in donors undergoing hepatectomy, and no significant difference was found between the solutions regarding postoperative renal outcomes and creatinine levels.^17^ With an increase in the number of randomized comparative studies in this field, more extensive information can be obtained.

### Study Limitations

One of this study’s limitations was that, as the experiment used a rat model, it was not possible to perform non-invasive hemodynamic monitoring. While it was possible to monitor parameters such as cardiac output and extravascular lung fluid volume in studies conducted on swine, it was not possible to monitor these parameters in the present study, in which we applied fluid resuscitation.^18^ Another limitation is that we did not compare the acid-base balance between the groups. Hyperchloremic metabolic acidosis has been reported in experimental animals that were administered NS in high volumes. However, since the volume of crystalloids applied was the same as the volume of bleeding, there was no incidence of high-volume infusion in our study.

## Conclusion

This study suggests that NS and RL can be safely used in the early phase of traumatic hemorrhagic shock up to the time when blood is available for transfusion. This conclusion was reached considering the improvement in the hemodynamic findings of hypovolemic shock, a reduction in apoptosis, and the improvement of the symptoms in favor of coagulopathy in bedside coagulation tests. However, there remains a need for further studies to reveal the differences specific to different resuscitation fluids.

## Figures and Tables

**Table 1:**
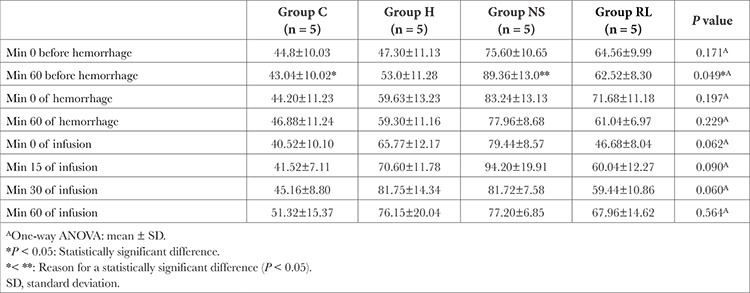
Comparison of Respiratory Rate Values Between Groups

**Table 2:**
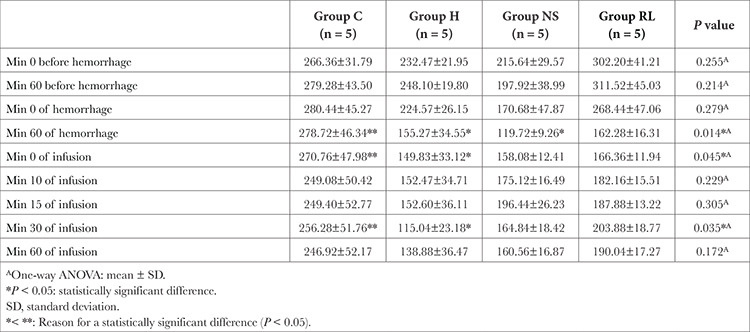
Comparison of Heart Rate Values Between Groups

**Table 3:**
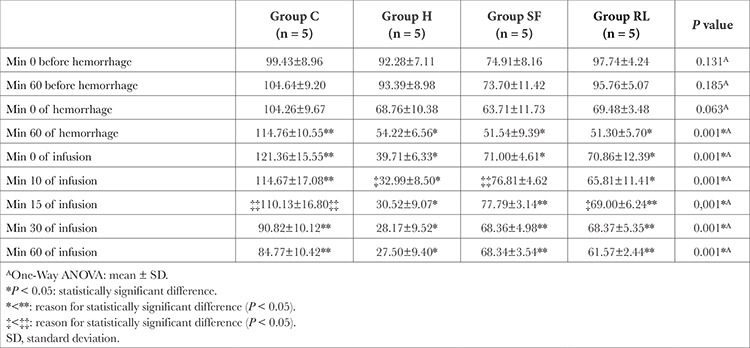
Comparison of Mean Blood Pressure Values Between Groups

**Table 4:**
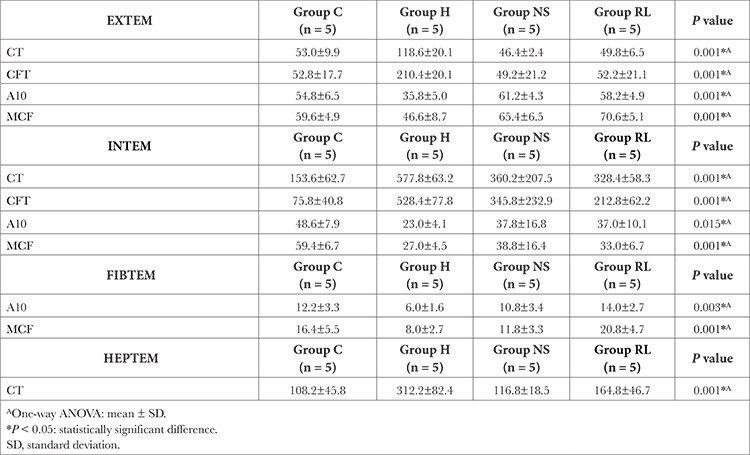
Comparison of EXTEM, INTEM, and FIBTEM Values Between Groups

**Table 5:**
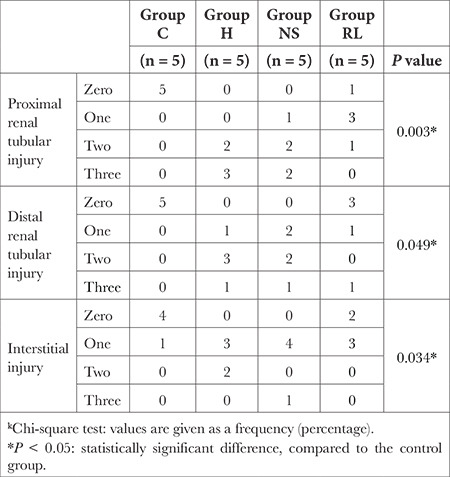
Comparison of Renal Histopathological Evaluation Scores Between Groups

**Table:**
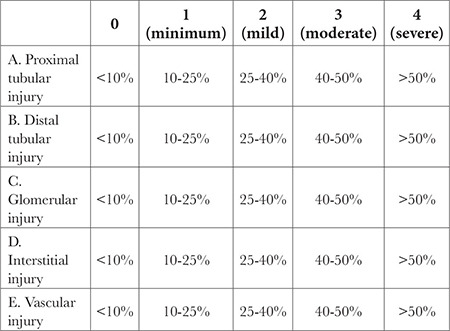


**Figure 1:**
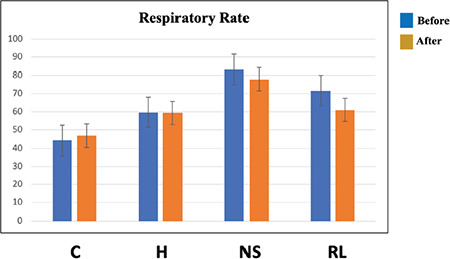
Comparison of respiratory rates recorded while inducing hemorrhage in the experiment animals. C, control group; H, hemorrhage group; NS, normal saline group; RL, Ringer’s lactate group.
